# Tungsten-Modulated Molybdenum Selenide/Graphene Heterostructure as an Advanced Electrode for All-Solid-State Supercapacitors

**DOI:** 10.3390/nano11061477

**Published:** 2021-06-02

**Authors:** Qixian Liu, Jing Ning, Haibin Guo, Maoyang Xia, Boyu Wang, Xin Feng, Dong Wang, Jincheng Zhang, Yue Hao

**Affiliations:** 1 The State Key Discipline Laboratory of Wide Band Gap Semiconductor Technology, Xidian University, Xi’an 710071, China; lqx@stu.xidian.edu.cn (Q.L.); guohaibin0719@163.com (H.G.); maoyangxia@126.com (M.X.); wangbyxd@stu.xidian.edu.cn (B.W.); xfengk@126.com (X.F.); chankwang@xidian.edu.cn (D.W.); jchzhang@xidian.edu.cn (J.Z.); yhao@xidian.edu.cn (Y.H.); 2 Shaanxi Joint Key Laboratory of Graphene, Xidian University, Xi’an 710071, China; 3 Xidian-Wuhu Research Institute, Wuhu 241000, China

**Keywords:** tungsten-modulated, molybdenum selenide/graphene, all-solid-state supercapacitors

## Abstract

Transition metal dichalcogenides (TMDs) have attracted widespread attention due to their excellent electrochemical and catalytic properties. In this work, a tungsten (W)-modulated molybdenum selenide (MoSe_2_)/graphene heterostructure was investigated for application in electrochemistry. MoSe_2_/graphene heterojunctions with low-doped W compositions were synthesized by a one-step hydrothermal catalysis approach. Based on the conducted density functional theory (DFT) calculations, it was determined that inserting a small amount of W (≈5%) into the MoSe_2_/graphene heterostructure resulted in the modification of its lattice structure. Additionally, an increase in the distance between layers (≈8%) and a decrease in the adsorption energy of the potassium ions (K^+^) (≈−1.08 eV) were observed following W doping. Overall, the electrochemical performance of the MoSe_2_/graphene hybrid was enhanced by the presence of W. An all-solid-state supercapacitor device prepared using electrodes based on the W-doped MoSe_2_/graphene composite achieved excellent capacitance of 444.4 mF cm^−2^ at 1 mV s^−1^. The results obtained herein revealed that the MoSe_2_/graphene hybrid exhibiting low W composition could be valuable in the field of energy storage and isoelectronic doping of TMDs.

## 1. Introduction

Owing to the rapid decrease in fossil fuels and increasing energy demand, the development of simple and effective energy sources is essential [1,2,3]. Supercapacitors, which are considered to be excellent electrochemical energy sources, exhibit good power and energy density, high cycling life as well as rapid charge and discharge rate. Hence, they are regarded as efficient and reliable energy storage and switch devices [4,5,6].

Recently, transition metal dichalcogenides (TMDCs) have been used as active electrode materials in supercapacitors due to their unique properties, including a large specific surface area, layered structure, electrical conductivity, as well as thermal and chemical stabilities [7,8,9]. Among different TMDCs, molybdenum selenide (MoSe_2_) is a particularly important material displaying a large layer spacing (0.646 nm), which decreases the energy barrier for adsorption/desorption of charged ions [10,11,12]. Nevertheless, MoSe_2_ tends to deform during the ion exchange process. Other limitations of this material include low electrical conductivity and cycling stability [13,14]. Transition metal chalcogenides/graphene hybrids have been considered in several studies [15,16,17,18] to improve the specific capacitance and cycle life of TMDCs. Notably, isoelectronic doping of TMDCs can result in the formation of ternary or quaternary alloys, which leads to changes in the physical and chemical properties of the materials (e.g., energy band, electrical, and optical properties) [19,20,21]. Previous reports show electrochemical enhancement and changes in the surface area and conductivity following isoelectronic doping [22,23].

In this work, we conducted a hydrothermal synthesis of a MoSe_2_/graphene heterojunction containing a low content of tungsten (W). The prepared material could be used as an electrode in supercapacitors and exhibited excellent potassium ion (K^+^) storage performance. Density functional theory (DFT) calculations were used to analyze the effect of different W doping concentrations on the interlayer distance and K^+^ adsorption energy of the MoSe_2_/graphene heterojunction. It was determined that the W-doped MoSe_2_ electrode based on a 3D graphene framework displayed higher K^+^ storage capacity and enhanced stability.

## 2. Experimental Methods

### 2.1. Density Functional Theory 

The first principle calculation was based on DFT using the pseudopotential plane-wave method in the Cambridge Sequential Total Energy Package (CASTEP). The exchange-correlation potential was described by the Perdew–Burke–Ernzerhof (PBE) functional using generalized gradient approximation (GGA). The plane-wave energy cutoff was set to 380 eV, and the Monkhorst–Pack method with G-centered 2 × 2 × 1 K-points mesh was used for the Brillouin zone. The convergence criteria for the energy and force calculations were set to 2.0 × 10^−^^5^ eV per atom and 0.05 eV/Å, respectively. The vacuum space was set to 20 Å.

### 2.2. Growth of Graphene 

The nickel foam was sonicated in ammonium persulfate solution for 30 min. The foam was subsequently washed with acetone, ethanol, and deionized water. The substrate for graphene growth was obtained following drying the foam under argon. Following the evacuation of the quartz tube furnace to vacuum, the nickel foam was heated from room temperature to 1000 °C under 15 cm^3^ min^−1^ hydrogen atmosphere. During this period, the vacuum value is about 10–100 Pa, and the heating time was 40 min. Then 50 cm^3^ min^−1^ of methane was introduced into the tube to grow graphene at 1000 °C for 1 h under a mixed atmosphere. Finally, the graphene sample was quickly cooled at a rate of 200 degrees per minute.

### 2.3. Preparation of a W-Doped MoSe_2_/Graphene Heterostructure and W-Doped MoSe_2_ Powder 

Point three grams of Na_2_MoO_4_**·**2H_2_O, 0.3 g of (NH_4_)_5_H_5_[H_2_(WO_4_)_6_]·H_2_O, and 0.2 g of Se were mixed with 5–10 mL of N_2_H_4_**·**H_2_O and stirred for 15 min. (All chemicals are purchased from Kequan Laboratory Co., Ltd., Xi’an, China) Fifty milliliters of deionized water was added, and the solution was stirred for 15 min. After mixing, the mixture was divided into two equal parts and transferred to two 60 mL Teflon-lined stainless steel autoclaves. The prepared nickel foam/graphene was subsequently placed in one of them. The mixture was heated to 160 °C for 24 h. The sample containing the nickel foam/graphene was then taken out and washed several times with deionized water and ethanol. The sample was dried at 80 °C for 24 h under a vacuum. Ten milliliters of the reaction solution was added into another container, centrifuged, and ultrasonicated. At the same time, different Mo: W ratios (1:1, 1:2, 2:1) and various temperatures (140 °C, 150 °C, 170 °C, 180 °C) were investigated to study the impact on the performance of composite electrodes.

### 2.4. Characterization of Materials

The W-doped MoSe_2_/graphene nanostructures were investigated by SEM (Quanta 600FEG, FEI, Hillsboro, OH, USA). The crystallinity of the materials was evaluated by high-resolution XRD (D8 Discovery, Bruker, Berlin, Germany) analysis in the Bragg (reflection) geometry with a pure Cu k_α1_ radiation (wavelength; λ; 1.54056 Å). The Raman spectrum was obtained with a Raman spectroscopy system (Lab Ram HR 800, Horiba JY, Kuoto, Japan) with an Ar^+^ laser (514 nm wavelength) as the excitation source. The elemental analysis of the composite was conducted by XPS (ESCALAB, 250Xi, ThermoFisher Scientific, Waltham, MA, USA).

### 2.5. Preparation of the Electrolyte

Five grams of PVA (polyvinyl alcohol ) was added to 35 mL of deionized water. The solution was stirred in a water bath at 80 °C until the PVA was completely dissolved. Subsequently, 5 g of KOH was dissolved in 5 mL of deionized water. The KOH solution was slowly added to the solution of PVA and stirred.

### 2.6. Electrochemical Analysis 

A three-electrode test system was used for the electrochemical measurements. The system was composed of an active material as the working electrode, Ag/AgCl as the reference electrode, platinum mesh as the counter electrode, and 3 M KOH as the electrolyte. For the electrochemical measurements of the all-solid-state supercapacitors, the electrodes and cellulose membrane were stacked in a sequence. The active material was in direct contact with the current collector. The electrolyte employed for the analysis was PVA–KOH. CV was conducted at a scan rate of 1 to 50 mV s^−1^. Constant current charging/discharging was performed at a current density of 0.5 to 8 mA cm^−2^. EIS was conducted in the frequency range of 0.01 Hz to 100 kHz with a disturbance of 5 mV (rms) under open-circuit voltage. 

For the CV curves, the specific capacitance values were calculated using Equation (1):

(1) $$C_{s}=\frac{\int I\left(V\right)\mathrm{dv}}{vS\Delta V}\;(\mathrm{mF}\;{\mathrm{cm}}^{-2})$$

where S refers to the working area of the supercapacitor (cm^2^), V indicates the scan rate (vs^−1^), and △V is the potential window (V).

For the GCD curves, the specific capacitance was calculated according to Equation (2):

(2) $$C_{S}=\frac{I\;\Delta t}{S\;\Delta V}\;(\mathrm{mF}\;{\mathrm{cm}}^{-2})$$

where I is the discharge current (mA) and △t denotes the discharge time (s).

The energy density and power density of the supercapacitor cell were obtained using Equations (3) and (4):

(3) $$E=\frac{C_{s}\Delta V^{2}}{2}\;(\mathrm{mWh}\;{\mathrm{cm}}^{-2})$$

(4) $$P=\frac{E}{\Delta t}\;(\mathrm{mW}\;{\mathrm{cm}}^{-2})$$

where E refers to the energy density (mWh cm^−2^), P indicates the power density (mW cm^−2^), and Δt (s) is the total discharge duration.

## 3. Results and Discussion

**Figure 1:**
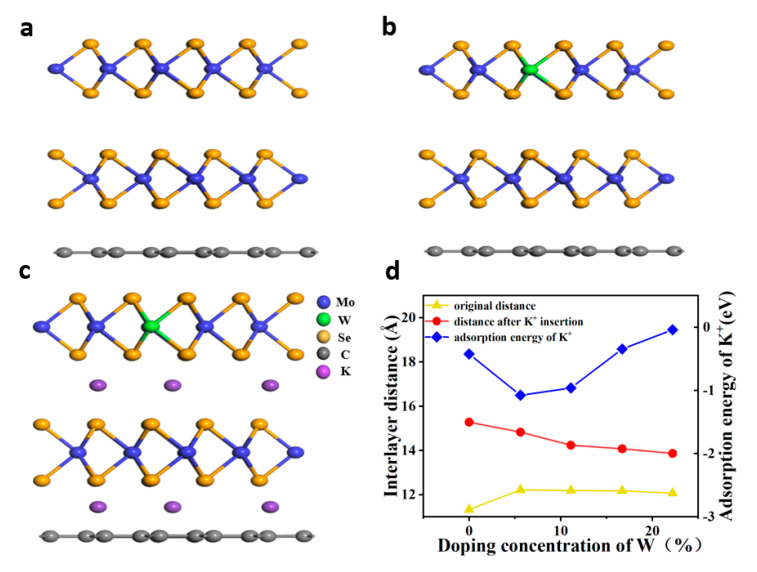
(**a**) Side view of MoSe_2_/graphene. (**b**) Side view of MoSe_2_/graphene containing 5.6% W. (**c**) Side view of MoSe_2_/graphene containing 5.6% W and intercalated K^+^ ions. Orange balls represent Se atoms, blue balls indicate Mo atoms, the green ball refers to the W atom, and purple balls show potassium ions. (**d**) Interlayer distance of pure MoSe_2_/graphene and MoSe_2_ /graphene doped with different concentrations of W, interlayer distance in the presence of K^+^ intercalation in MoSe_2_/graphene and MoSe_2_/graphene doped with different concentrations of W, adsorption energy of K^+^ on MoSe_2_/graphene and MoSe_2_/graphene doped with different concentrations of W.

The structures of MoSe_2_/graphene containing low W contents are illustrated in Figure 1a. W atoms were used to replace Mo in the 3 × 3 × 1 supercell of MoSe_2_. When the Mo atoms were substituted by W, the doping concentration of W was 5.6%, which was consistent with the results obtained by X-ray photoelectron spectroscopy (XPS) (5%). Figure 1d shows the interlayer distance and K^+^ adsorption energy of MoSe_2_/graphene doped with different concentrations of W (5.6%, 11.1%, 16.7%, and 22.2%). Compared with pure MoSe_2_/graphene, MoSe_2_/graphene doped with W at a concentration of 5.6% exhibited a larger interlayer distance (≈ 8%, 12.22 Å) and smaller K^+^ adsorption energy (−1.08 eV). This facilitated the process of K^+^ adsorption/extraction. The adsorption energy of K ions ($E_{a}$) is determined by Equation (5):

(5) $$E_{a}=\frac{E_{\mathrm{total}\;\mathrm{energy}\;\mathrm{after}\;\mathrm{inserting}\;\mathrm{Kions}}-{\;E}_{\mathrm{total}\;\mathrm{energy}\;\mathrm{before}\;\mathrm{inserting}\;K\;\mathrm{ions}}-{\;\mathrm{nE}}_{K\;\mathrm{ions}}}{n}$$

where, the insertion energy of K ions between different layers at different W doping concentrations is shown in Figure S1. The adsorption energy of K ions between W-doped MoSe_2_ and graphene is higher than that between W-doped MoSe_2_. This may be the interlayer distance between graphene and W-doped MoSe_2_(≈ 5.6 Å) is smaller than that between tungsten-doped molybdenum selenide, making it more difficult for potassium ions to insert/detach.

**Figure 2:**
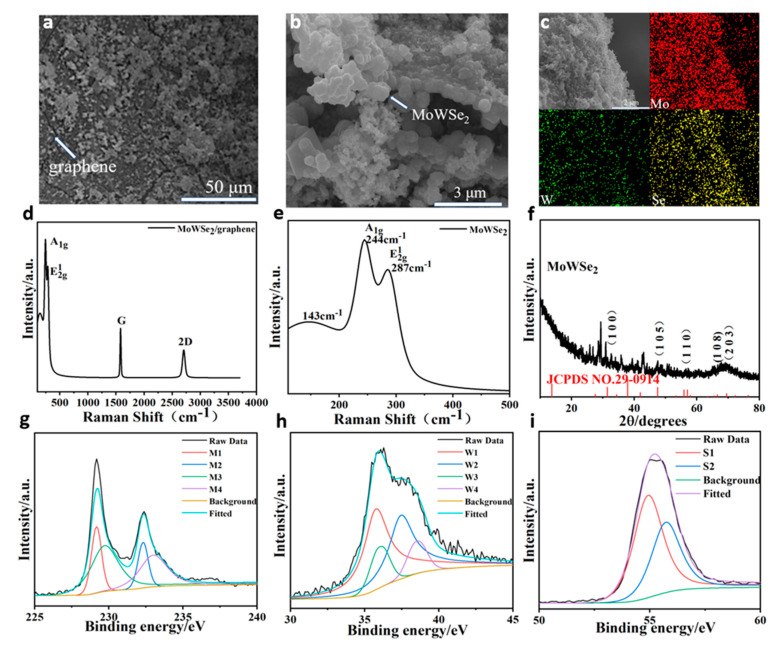
(**a–c**) SEM images of W-doped MoSe_2_/graphene and EDS images of W-doped MoSe_2_/graphene. (**d**,**e**) Raman spectra of W-doped MoSe_2_/graphene. (**f**) XRD patterns of W-doped MoSe_2_**.** (**g**–**i**) XPS survey spectra of W-doped MoSe_2_ (Mo 3d, W 4f, and Se 3d).

Scanning electron microscopy (SEM) and energy dispersive spectrometry (EDS) were used to characterize the W-doped MoSe_2_/graphene nanostructures (Figure 2a–c). It was determined that the graphene films prepared by low-pressure chemical vapor deposition (LPCVD) were stacked on the framework of the nickel foam (Figure 2a), while the W-doped MoSe_2_ particles grown on graphene assembled to form nanoballs and nanoflowers (Figure 2b,c). The composite structures of W-doped MoSe_2_/graphene closely interacted with each other. The potential structures of W-doped MoSe_2_ and graphene are demonstrated in Figure 2b. Moreover, the EDS images of W-doped MoSe_2_/graphene nanostructures revealed the presence of Mo, W, and Se. Through SEM, we observed that the presence of graphene not only provides a framework for the growth of W-doped MoSe_2_ materials but also enhances the electrical conductivity and contact area of the composite material, thereby providing more active sites. The nanoflower structure of W-doped MoSe_2_ is uniformly grown on the graphene framework, providing a larger surface area and a shorter ion channel during charge and discharge. The two materials complement each other and enhance the electrochemical performance of the composite material.

The outcomes of the Raman spectroscopy analysis of W-doped MoSe_2_/graphene nanostructures are shown in Figure 2d,e. The peaks at 1580.7 and 2705.0 cm^−1^ corresponded to the G and 2D bands of graphene, respectively. In addition, the signals at 244.2 and 287.5 cm^−1^ were attributed to the A_1g_ and E_2g_^1^ modes, correspondingly. A slight shift of the peaks to the lower wavenumbers compared to those of pure MoSe_2_ confirmed the successful doping of W in MoSe_2_ [24]. The spectral data and SEM images of the raw graphene are shown in Figures S2 and S3. The results of the X-ray diffraction (XRD) evaluation are demonstrated in Figure 2f. The characteristic peaks at 30.9°, 47.5°, 55.5°, 66.6°, and 69.6° corresponded to the (1 0 0), (1 0 5), (1 1 0), (1 0 8), and (2 0 3) crystal planes of the W-doped MoSe_2_ nanostructure, respectively. The detected peaks were comparable to those of pure MoSe_2_; however, a small shift to lower values was noted. The particle size of the powdered samples was not uniform enough, and there were some by-products in the hydrothermal synthesis, resulting in a lot of XRD data miscellaneous peaks, such as Mo_3_Se_4_, Mo_15_Se_19_, and Se rings [25]. Therefore, we tested W-doped MoSe_2_/graphene/Ni foam electrode samples. The electrode was washed several times with ethanol and deionized water and dried in a vacuum oven at 60 °C for 12 h, in order to obtain a well-crystallized product. The prepared materials are shown in Figure S4. We observe peaks at 25.73°, 31.8°, 33.6°, 76.3°, 81.3° corresponding to the (0 0 4), (1 0 0), (1 0 2), (2 0 5) and (2 0 6) crystal orientations of MoSe_2_(PDF#29-0914). A slight shift in the peaks to the lower wavenumbers compared to those of pure MoSe_2_ confirmed doping of W in MoSe_2_. The peaks at 43.3°, 51.8°, and 81.4° of graphene position corresponding to the (1 0 0), (1 0 2), and (1 1 2) crystal (PDF#41-1487). Furthermore, XPS was employed to investigate the elemental composition of the W-doped MoSe_2_ nanostructure (Figure 2g–i). The Mo 3d peaks attributed to Mo^+4^ 3d 5/2 and Mo^+4^ 3d 3/2 were detected at 229.2 and 232.4 eV, respectively, and were comparable to those of bulk MoS_2_ and MoSe_2_. Similarly, the W 4f peaks could be divided into W 4f 7/2 and W 4f 5/2 at the binding energies of 35.8 and 36.3 eV (W1 and W2, respectively) (Figure 2h). The ratios of the characteristic peak areas of different elements to the corresponding atomic sensitivity factors were normalized to calculate that the doping concentration of W atomic percentage of the element is about 5.6%. It is noted that the peaks at 230 eV (M3) and 233 eV (M4) can be separately attributed to +3 and +6 oxidation state of Mo, due to the oxidation in the hydrothermal synthesis process. The peaks of W1 and W2 shifted to the direction of high energy by about 2 eV. This may be due to the loss of electrons from W during the formation of Mo-W bonding. In addition, the peaks at higher binding energy 37 eV(W3) and 38 eV(W4) can be assigned to the bonding of Mo-W and tungsten oxides. Owing to the spin–orbit coupling, the 3d peaks of Se (Figure 2i) could be assigned to the 3d 5/2 and 3d 3/2 states at 54.9 and 55.7 eV, respectively [26].

**Figure 3:**
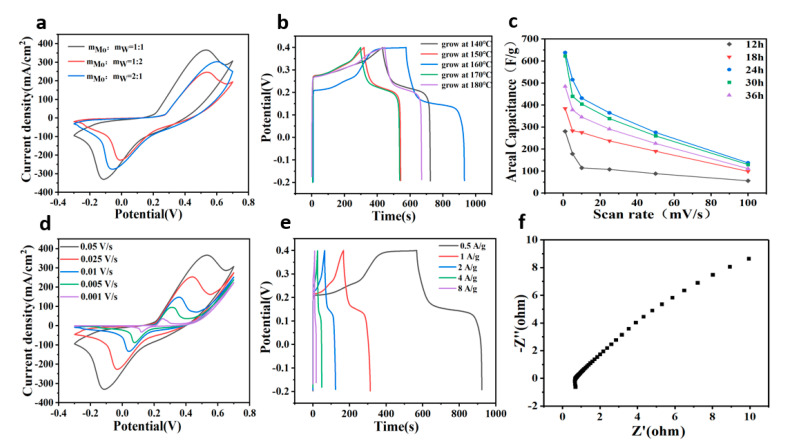
(**a**) CV curves of W-doped MoSe_2_/graphene electrodes with a different molar mass ratio at 50 mV s^−1^. (**b**) GCD curves of W-doped MoSe_2_/graphene electrodes at various growth temperatures at 0.5 A g^−1^. (**c**) Areal capacitance of W-doped MoSe_2_/graphene electrodes at a different growth time. (**d**) CV curves of W-doped MoSe_2_/graphene electrodes at different scan rates. (**e**) GCD curves of W-doped MoSe_2_/graphene electrodes at various current densities. (**f**) Nyquist plots of W-doped MoSe_2_/graphene electrodes.

The electrochemical performance of W-doped MoSe_2_/graphene electrodes was evaluated by cyclic voltammetry (CV), galvanostatic charge–discharge (GCD), and electrochemical impedance spectroscopy (EIS) using 3 M KOH as the electrolyte. Different ratios of raw materials (mMo:mW = 1:1, 1:2, and 2:1), growth time (12, 18, 24, 30, and 36 h), and temperature (140 °C, 150 °C, 160 °C, 170 °C, and 180 °C) were tested to achieve the most optimal conditions for the growth the W-doped MoSe_2_/graphene heterostructure. Figure 3a illustrates the CV curves at a scan rate of 50 mV s^−1^. The largest area of mMo:mW = 1:1 under the CV curve of the electrode indicated the highest capacitance. Figure 3b compares different GCD curves at a growth temperature from 140 °C to 180 °C at 0.5 A g^−1^. The electrode grown at 160 °C displayed a longer discharge time, suggesting higher capacitance. Moreover, varying the growth time of the electrode demonstrated that the best results were achieved after 24 h. The material obtained after this time exhibited the largest capacitance (Figure 3c). Figure 3d shows the CV curves of W-doped MoSe_2_/graphene electrodes in the voltage range from −0.3 to 0.7 V at various scan rates. The presence of obvious symmetric redox peaks indicated the occurrence of a typical Faradaic process involving oxidation/reduction reactions. Similar shapes and broadening peaks were also be observed at higher scan rates, implying good performance. The GCD measurements were performed between −0.2 and 0.4 V at current densities ranging from 0.5 to 8 A g^−1^ (Figure 3e). W-doped MoSe_2_/graphene showed capacitance of 296, 245, 197, 155, and 114 F g^−1^ at a current density of 0.5, 1, 2, 4, and 8 A g^−1^, respectively. The EIS data summarized in Figure 3f suggested that W-doped MoSe_2_/graphene exhibited series resistance of 0.67 ohm and contact resistance of 0.2 ohm, which accelerated the electrostatic adsorption of ions. According to Figure 3d, the CV curve shows symmetric redox peaks. This indicates that there were non-Faraday and Faraday processes in the composite electrode. The Faraday process of adsorption/desorption of potassium ions at the active sites on the surface and the Faraday process of oxidation/reduction occurred in the internal nanostructure. The possible reactions are expressed by the following equations:

Mo(W)Se_2_ + K^+^ + e^−^ → Mo(W)Se_2_-K (surface process) (6)

Mo(W)Se_2_ +K^+^ +e^−^ → KMo(W)Se_2_ (redox process) (7)

**Figure 4:**
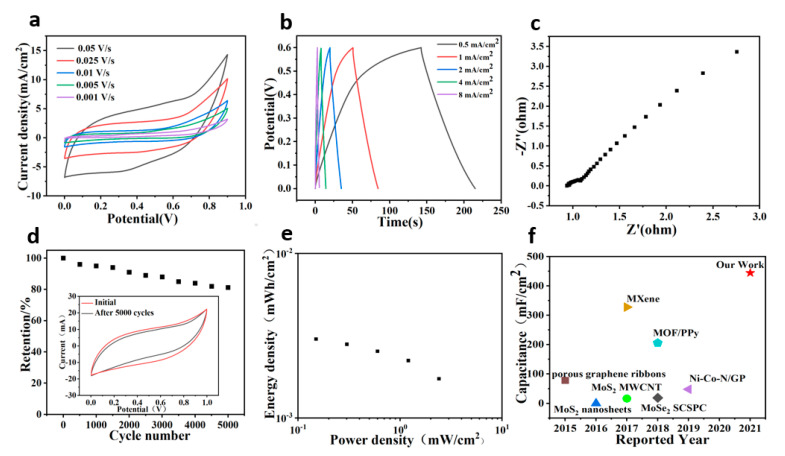
(**a**) CV curves of the all-solid-state supercapacitor based on W-doped MoSe_2_/graphene at different scan rates. (**b**) GCD curves of the all-solid-state supercapacitor based on W-doped MoSe_2_/graphene at various current densities. (**c**) Nyquist plots of the all-solid-state supercapacitor based on W-doped MoSe_2_/graphene. (**d**) Cycle lifetime of the all-solid-state supercapacitor based on W-doped MoSe_2_/graphene at 500 mV s^−1^. (**e**) Ragone plot of the all-solid-state supercapacitor based on W-doped MoSe_2_/graphene. (**f**) Plot of the gravimetric capacitance of W-doped MoSe_2_/graphene compared to other previously reported 2D electrode materials.

A symmetrical all-solid-state supercapacitor was assembled using a polyvinyl alcohol (PVA)–KOH electrolyte and W-doped MoSe_2_/graphene electrode to investigate its energy storage behavior. Figure 4a demonstrates the CV curves of the all-solid-state supercapacitor in a voltage window of 0–0.9 V. No redox peaks were detected at different scanning rates with symmetrical cathode and anode, which indicated equal capacitance during charge and discharge. This implied that the all-solid-state supercapacitor was a Faraday quasi-capacitor. The capacitance was established to be 444.4, 266.7, 211.1, 182.2, and 157.8 mF cm^−2^ at a scanning rate of 1, 5, 10, 25, and 50 mV s^−1^, respectively. Figure 4b shows the GCD curve of the all-solid-state supercapacitor. The capacitance was equal to 60.5, 56.2, 51.0, 44.7, and 34.7 mF cm^−2^ at a current density of 0.5, 1, 2, 4, and 8 mA cm^−2^. The charge and discharge curves were roughly equal, indicating that the supercapacitor exhibited excellent energy storage properties. Furthermore, Figure 4c demonstrates the Nyquist plots of the all-solid-state supercapacitor based on W-doped MoSe_2_/graphene. The equivalent series resistance of the supercapacitor was determined at 0.9 ohm, while the charge transfer resistance was approximately 0.5 ohm. Figure 4d shows the cycle lifetime of the all-solid-state supercapacitor based on W-doped MoSe_2_/graphene at 500 mV s^−1^. Notably, 81.3% of the capacitance was retained after 5000 cycles. The SEM of the device after 5000 cycles was tested shown in Figure S5. It can be observed that the active material was reduced, part of it falls off, and there were cracks on the surface to expose the graphene. This was also the reason for the reduced capacitance and performance of the device after multiple cycles. The Ragone plot of the all-solid-state supercapacitor based on Gra/W-doped MoSe_2_ is illustrated in Figure 4e. The W-doped MoSe_2_/graphene all-solid-state supercapacitor exhibited an energy density of 0.003 mWh cm^−2^ at a power density of 0.15 mW cm^−2^. Compared to other previously reported all-solid-state supercapacitors, the electrode based on W-doped MoSe_2_/graphene heterostructure showed enhanced properties (Figure 4f) [27,28,29,30,31,32,33]. The combined action of the W-doped MoSe_2_/graphene heterostructure and the all-solid-state electrolyte resulted in excellent electrochemical performance.

## 4. Conclusions

In conclusion, MoSe_2_/graphene heterostructures containing low contents of W were successfully prepared by a catalytic hydrothermal method. DFT calculations were used to evaluate the characteristics of W-doped MoSe_2_/graphene, which exhibited a large interlayer distance and low K^+^ adsorption energy. This facilitated electrochemical reactions. Moreover, electrochemical investigations demonstrated that all-solid-state supercapacitors based on the MoSe_2_/graphene heterostructure displayed excellent supercapacitor performance. Specific capacity of 444.4 mF cm^−2^ was achieved at a scanning rate of 1 mV s^−1^. Notably, 81.3% of capacitance was retained after 5000 cycles at 500 mV s^−1^. 

## Data Availability

The data presented in this study are available from the corresponding author.

## Supplementary Materials

The following are available online at https://www.mdpi.com/article/10.3390/nano11061477/s1, Figure S1: Adsorption energy of K ions under different doping concentrations. Figure S2: Raman spectra of graphene. Figure S3: SEM image of graphene. Figure S4: (a) XRD spectrum of Graphene/Ni foam, (b) XRD spectrum of Mo:W = 1:1, (c) XRD spectrum of Mo:W = 1:2, (d) XRD spectrum of Mo:W = 2:1, (e) XRD spectrum of sample grown at 140 °C, (f) XRD spectrum of sample grown at 150 °C, (g) XRD spectrum of sample grown at 170 °C, (h) XRD spectrum of sample grown at 180 °C. Figure S5: SEM image of device after 5000 cycles.

## References

[1] Turner J.A. (1999). A realizable renewable energy future. Science.

[2] Lund H. (2007). Renewable energy strategies for sustainable development. Energy.

[3] Dincer I. (2000). Renewable energy and sustainable development: A crucial review. Renew. Sustain. Energy Rev..

[4] Zhang L.L., Zhao X.S. (2009). Carbon-based materials as supercapacitor electrodes. Chem. Soc. Rev..

[5] Wang Y., Shi Z., Huang Y., Ma Y., Wang C., Chen M., Chen Y. (2009). Supercapacitor devices based on graphene materials. J. Phys. Chem. C.

[6] Frackowiak E. (2007). Carbon materials for supercapacitor application. Phys. Chem. Chem. Phys..

[7] Cherusseri J., Choudhary N., Sambath Kumar K.S., Jung Y., Thomas J. (2019). Recent trends in transition metal dichalcogenide based supercapacitor electrodes. Nanoscale Horiz..

[8] Seman R.N.A.R., Azam M.A., Ani M.H. (2018). Graphene/transition metal dichalcogenides hybrid supercapacitor electrode: Status, challenges, and perspectives. Nanotechnology.

[9] Lin L., Lei W., Zhang S., Liu Y., Wallace G.G., Chen J. (2019). Two-dimensional transition metal dichalcogenides in supercapacitors and secondary batteries. Energy Storage Mater..

[10] Balasingam S.K., Lee J.S., Jun Y. (2015). Few-layered MoSe_2_ nanosheets as an advanced electrode material for supercapacitors. Dalton Trans..

[11] Zhao X., Cai W., Yang Y., Song X., Neale Z., Wang H., Sui J., Cao G. (2018). MoSe_2_ nanosheets perpendicularly grown on graphene with Mo–C bonding for sodium-ion capacitors. Nano Energy.

[12] Guo H., Ning J., Wang B., Feng X., Xia M., Wang D., Jia Y., Zhang J., Hao Y. (2021). Sodium ion-intercalated nanoflower 1T–2H MoSe_2_-graphene nanocomposites as electrodes for all-solid-state supercapacitors. J. Alloys Compd..

[13] Xia M., Ning J., Wang D., Feng X., Wang B., Guo H., Zhang J., Hao Y. (2021). Ammonia-assisted synthesis of gypsophila-like 1T-WSe_2_/graphene with enhanced potassium storage for all-solid-state supercapacitor. Chem. Eng. J..

[14] El-Mahalawy S.H., Evans B.L. (1977). Temperature dependence of the electrical conductivity and hall coefficient in 2H-MoS_2_, MoSe_2_, WSe_2_, and MoTe_2_. Phys. Stat. Sol..

[15] Lin T.W., Sadhasivam T., Wang A.Y., Chen T., Lin J., Shao L. (2018). Ternary composite nanosheets with MoS_2_/WS_2_/Graphene heterostructures as high-performance cathode materials for supercapacitors. Chem Electro Chem.

[16] Wang H., Feng H., Li J. (2014). Graphene and graphene-like layered transition metal dichalcogenides in energy conversion and storage. Small.

[17] Bissett M.A., Kinloch I.A., Dryfe R.A.W. (2015). Characterization of MoS_2_-graphene composites for high-performance coin cell supercapacitors. ACS Appl. Mater. Interfaces.

[18] Abraham A.M., Bharath G., Hai A., Banat F. (2019). Preparation of MoS_2_/graphene nanostructures and their supercapacitor and hydrogen evolution reaction (HER) performances. J. Phys. D.

[19] Zhao Y., Wang W., Li C., He L. (2017). First-principles study of nonmetal doped monolayer MoSe_2_ for tunable electronic and photocatalytic properties. Sci. Rep..

[20] Zhao Y., Wang W., Li C., He L. (2018). Tuning the magnetic properties of the monolayer MoSe_2_ by nonmetal doping: First-principles study. Solid State Commun..

[21] Ma Y., Dai Y., Guo M., Niu C., Lu J., Huang B. (2011). Electronic and magnetic properties of perfect, vacancy-doped, and nonmetal adsorbed MoSe_2_, MoTe_2_ and WS 2 monolayers. Phys. Chem. Chem. Phys..

[22] Sakthivel M., Ramaraj S., Chen S.M., Chen T.W., Ho K.C. (2019). Transition-metal-doped molybdenum diselenides with defects and abundant active sites for efficient performances of enzymatic biofuel cell and supercapacitor applications. ACS Appl. Mater. Interfaces.

[23] Falola B.D., Fan L., Wiltowski T., Suni I.I. (2017). Electrodeposition of Cu-doped MoS_2_ for charge storage in electrochemical supercapacitors. J. Electrochem. Soc..

[24] Zhang M., Wu J., Zhu Y., Dumcenco D.O., Hong J., Mao N., Deng S., Chen Y., Yang Y., Jin C. (2014). Two-dimensional molybdenum tungsten diselenide alloys: Photoluminescence, Raman scattering, and electrical transport. ACS Nano.

[25] Bhat K.S., Nagaraja H.S. (2019). Effect of isoelectronic tungsten doping on molybdenum selenide nanostructures and their graphene hybrids for supercapacitors. Electrochim. Acta..

[26] Ambrosi A., Sofer Z., Pumera M. (2015). 2H→ 1T phase transition and hydrogen evolution activity of MoS_2_, MoSe_2_, WS 2 and WSe_2_ strongly depends on the MX 2 composition. Chem. Commun..

[27] Pazhamalai P., Krishnamoorthy K., Mariappan V.K., Sahoo S., Manoharan S., Kim S.J. (2018). A high efficacy self-charging MoSe_2_ solid-state supercapacitor using electrospun nanofibrous piezoelectric separator with Ionogel electrolyte. Adv. Mater. Interfaces.

[28] Qi K., Hou R., Zaman S., Qiu Y., Xia B.Y., Duan H. (2018). Construction of metal–organic framework/conductive polymer hybrid for all-solid-state fabric supercapacitor. ACS Appl. Mater. Interfaces.

[29] Karade S.S., Dubal D.P., Sankapal B.R. (2017). Decoration of ultrathin MoS_2_ nanoflakes over MWCNTs: Enhanced supercapacitive performance through electrode to symmetric all-solid-state device. ChemistrySelect.

[30] Yang B., Hao C., Wen F., Wang B., Mu C., Xiang J., Li L., Xu B., Zhao Z., Liu Z. (2017). Flexible black-phosphorus nanoflake/carbon nanotube composite paper for high-performance all-solid-state supercapacitors. ACS Appl. Mater. Interfaces.

[31] Krishnamoorthy K., Pazhamalai P., Veerasubramani G.K., Kim S.J. (2016). Mechanically delaminated few layered MoS_2_ nanosheets based high performance wire type solid-state symmetric supercapacitors. J. Power Sources.

[32] Liu F., Zeng L., Chen Y., Zhang R., Yang R., Pang J., Ding L., Liu H., Zhou W. (2019). Ni-Co-N hybrid porous nanosheets on graphene paper for flexible and editable asymmetric all-solid-state supercapacitors. Nano Energy.

[33] Hu M., Li Z., Li G., Hu T., Zhang C., Wang X. (2017). All-solid-state flexible fiber-based MXene supercapacitors. Adv. Mater. Technol..

