# Supplementary material for: Tungsten-Modulated Molybdenum Selenide/Graphene Heterostructure as an Advanced Electrode for All-Solid-State Supercapacitors
Source: Nanomaterials (Basel). 2021 Jun 2;11(6):1477. doi: 10.3390/nano11061477 (PMC8228879; doi:10.3390/nano11061477)
Supplement: Supplementary file 1 [file nanomaterials-11-01477-s001.zip › nanomaterials-1217782-SI.pdf]

nanomaterials

Supplementary Materials

Tungsten-Modulated Molybdenum Selenide/Graphene Heterostructure as an Advanced Electrode for All-Solid-State Supercapacitors

Qixian Liu [1,2,3], Jing Ning [1,2,3,*], Haibin Guo [1,2,3], Maoyang Xia [1,2,3], Boyu Wang [1,2,3], Xin Feng [1,2,3], Dong Wang [1,2,3], Jincheng Zhang [1,2,3] and Yue Hao [1,2,3]

1 The State Key Discipline Laboratory of Wide Band Gap Semiconductor Technology, Xidian University, Xi'an 710071, China; lqx@stu.xidian.edu.cn (Q.L.); guohaibin0719@163.com (H.G.); maoyangxia@126.com (M.X.); wangbyxd@stu.xidian.edu.cn (B.W.); xfengk@126.com (X.F.); chankwang@xidian.edu.cn (D.W.); jchzhang@xidian.edu.cn (J.Z.); yhao@xidian.edu.cn (Y.H.)
2 Shaanxi Joint Key Laboratory of Graphene, Xidian University, Xi'an 710071, China
3 Xidian-Wuhu Research Institute, Wuhu 241000, China
* Correspondence: ningj@xidian.edu.cn

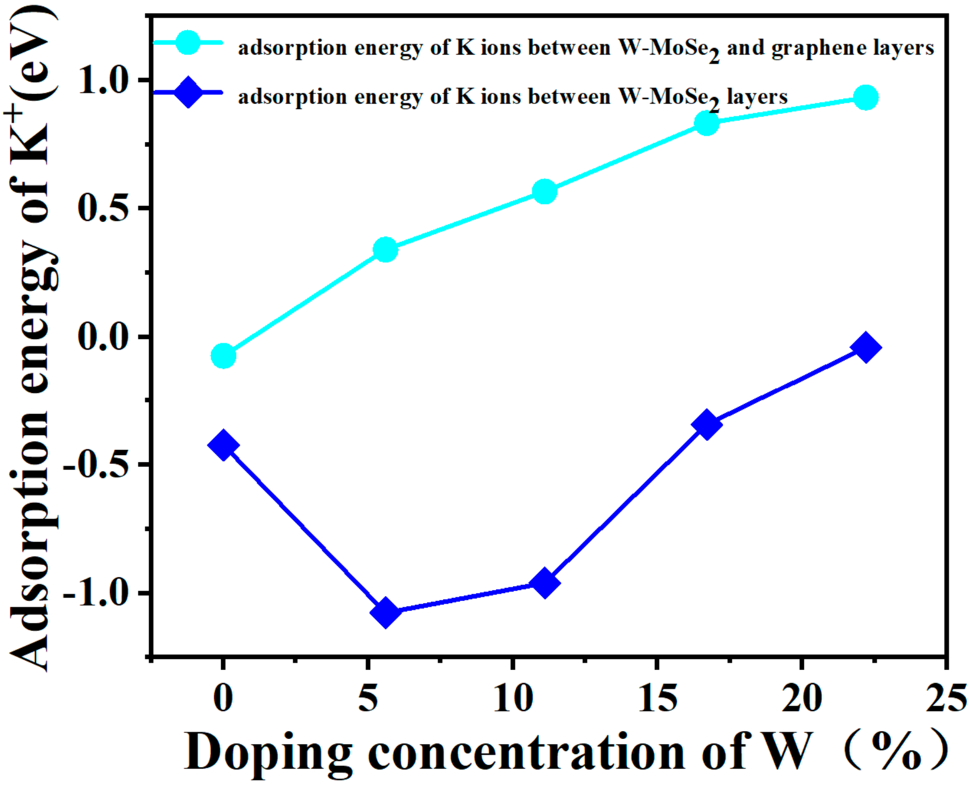

Figure S1. Adsorption energy of K ions under different doping concentrations.

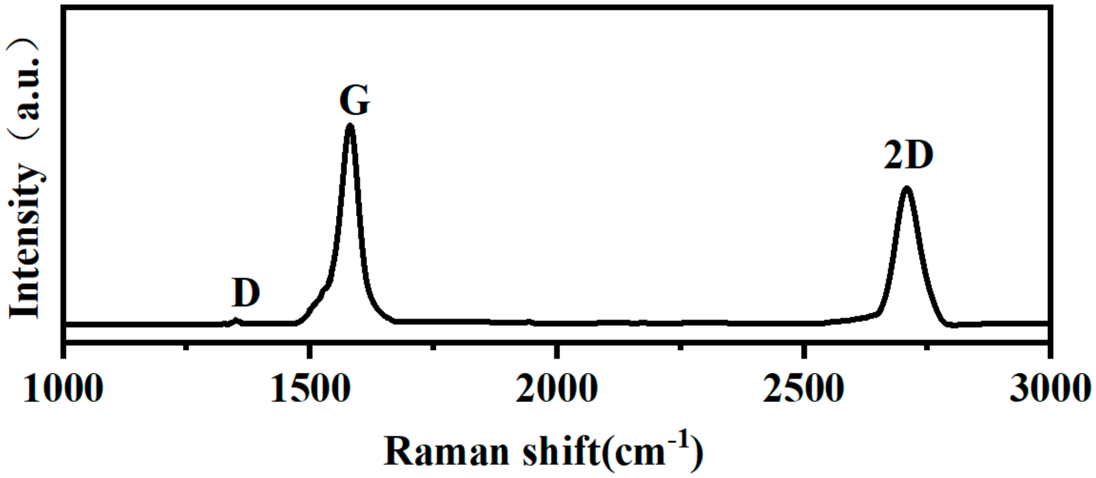

Figure S2. Raman spectra of graphene.

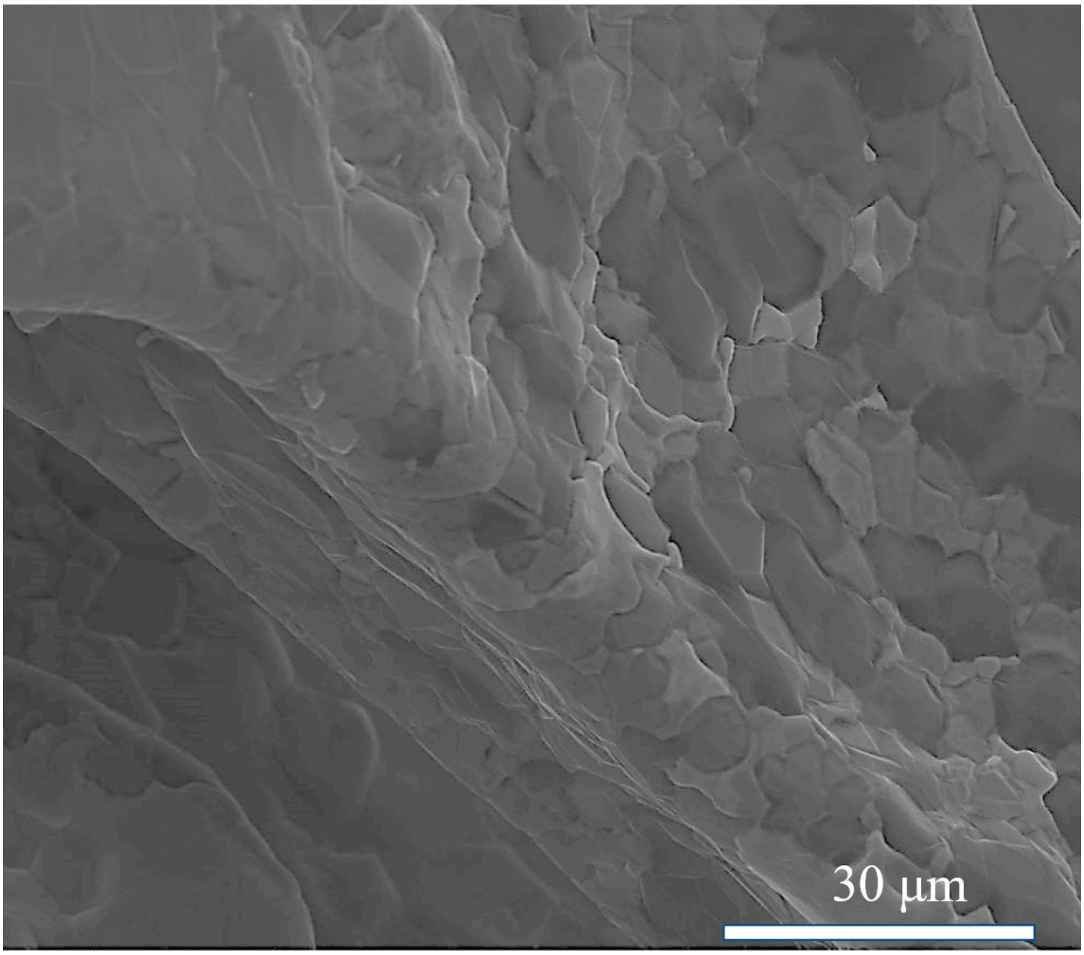

Figure S3. SEM image of graphene.

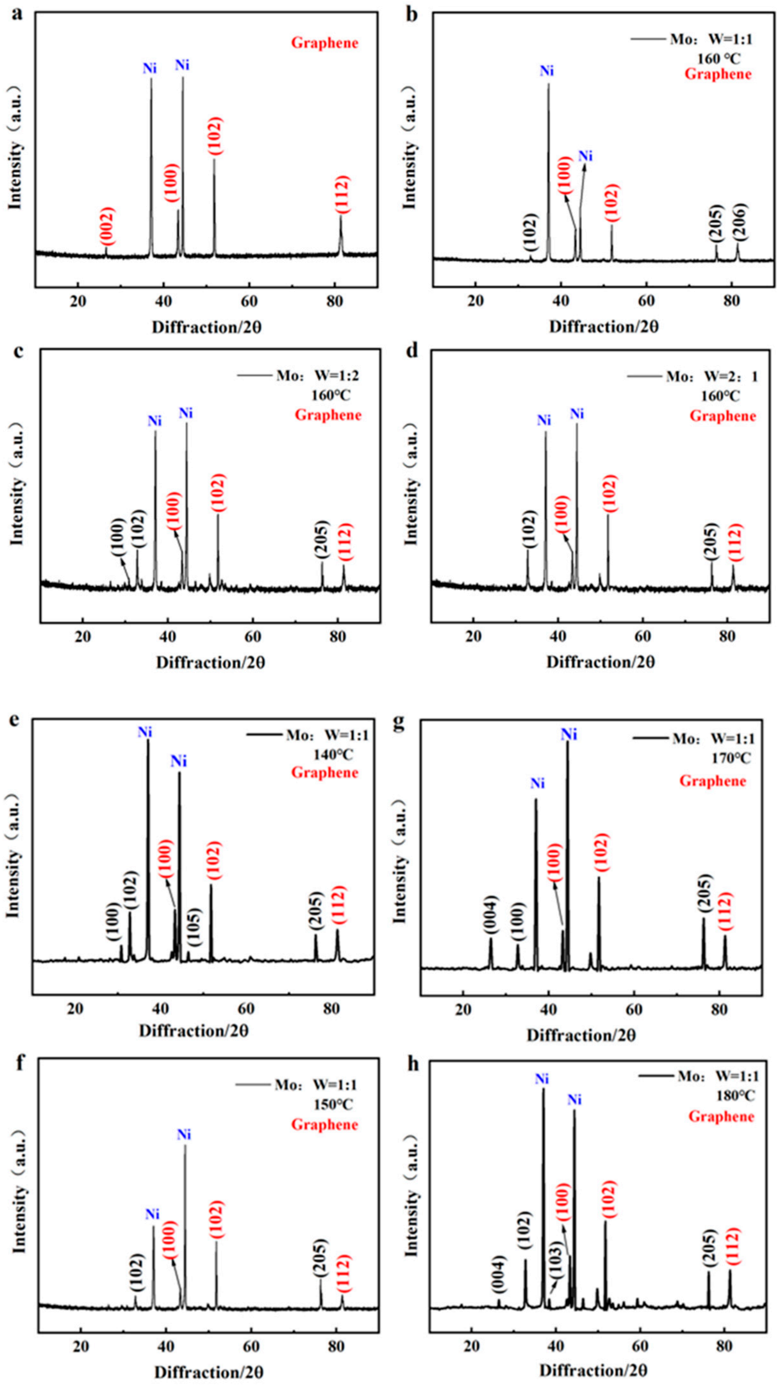

Figure S4. (a) XRD spectrum of Graphene/Ni foam, **(b)** XRD spectrum of Mo:W = 1:1, **(c)** XRD spectrum of Mo:W = 1:2, **(d)** XRD spectrum of Mo: W = 2:1, **(e)** XRD spectrum of sample grown at 140 °C, **(f)** XRD spectrum of sample grown at 150 °C, **(g)** XRD spectrum of sample grown at 170 °C**, (h)** XRD spectrum of sample grown at 180 °C.

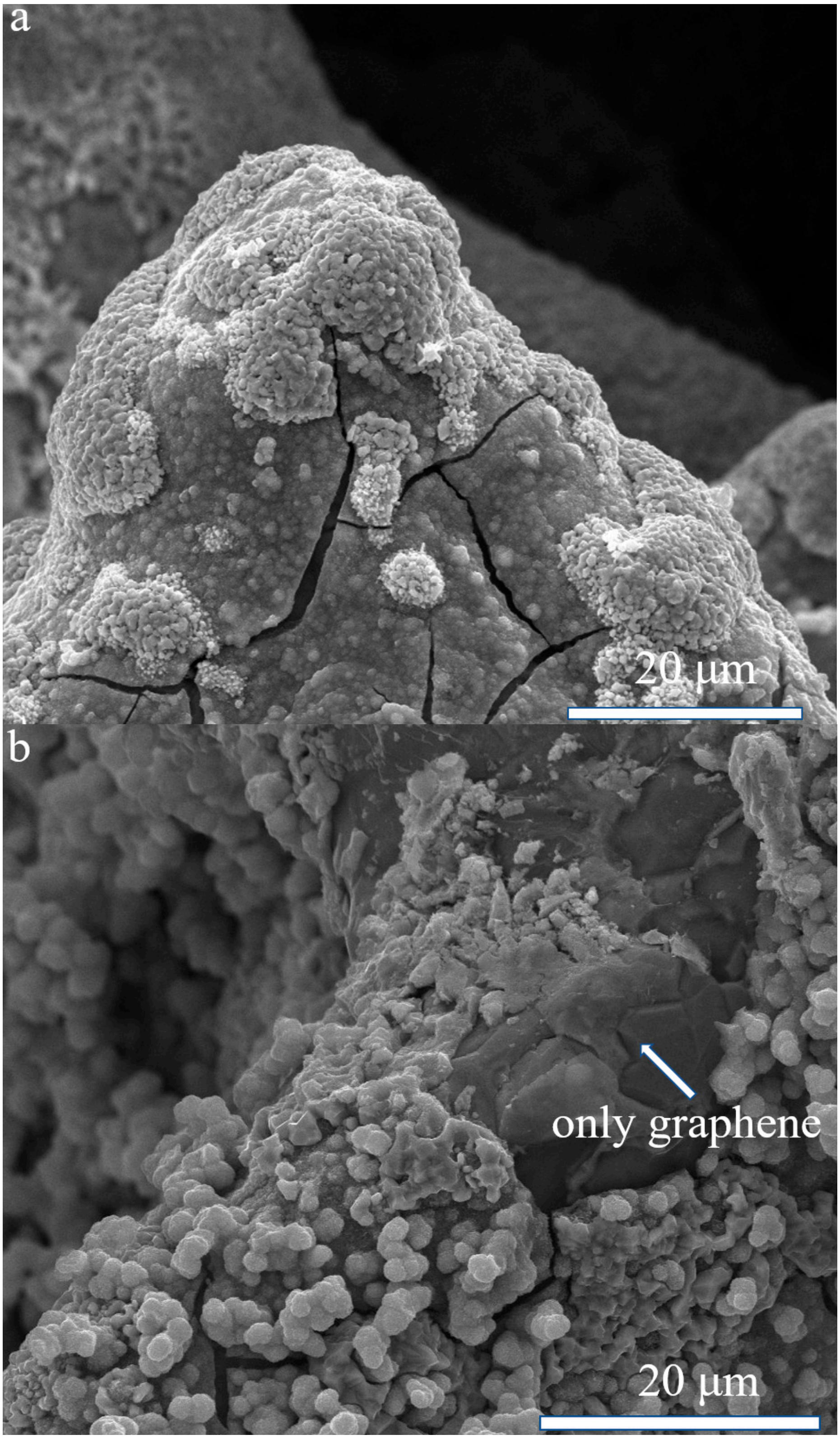

Figure S5. (a) SEM image of device after 5000 cycles, **(b)** The active material falls off and part of the graphene area is exposed after 5000 cycles.
